# Assessment of the impact of a personalised nutrition intervention in impaired glucose regulation over 26 weeks: a randomised controlled trial

**DOI:** 10.1038/s41598-024-55105-6

**Published:** 2024-03-05

**Authors:** Maria Karvela, Caroline T. Golden, Nikeysha Bell, Stephanie Martin-Li, Judith Bedzo-Nutakor, Natalie Bosnic, Pierre DeBeaudrap, Sara de Mateo-Lopez, Ahmed Alajrami, Yun Qin, Maria Eze, Tsz-Kin Hon, Javier Simón-Sánchez, Rashmita Sahoo, Jonathan Pearson-Stuttard, Patrick Soon-Shiong, Christofer Toumazou, Nick Oliver

**Affiliations:** 1 Department of Electrical and Electronic Engineering, Imperial College London, London, SW7 2AZ UK; 2 DnaNudge Ltd, Scale Space, Imperial College London, White City Campus, London, UK; 3 Centre for Population and Development (Ceped), French National Institute for Sustainable Development (IRD), and Paris University, Inserm ERL, 1244 Paris, France; 4 School of Public Health, Imperial College London, London, UK; 5 Department of Metabolism, Digestion and Reproduction, Faculty of Medicine, Imperial College London, London, UK

**Keywords:** Disease prevention, Nutrition, Public health

## Abstract

Dietary interventions can reduce progression to type 2 diabetes mellitus (T2DM) in people with non-diabetic hyperglycaemia. In this study we aimed to determine the impact of a DNA-personalised nutrition intervention in people with non-diabetic hyperglycaemia over 26 weeks. ASPIRE-DNA was a pilot study. Participants were randomised into three arms to receive either (i) Control arm: standard care (NICE guidelines) (n = 51), (ii) Intervention arm: DNA-personalised dietary advice (n = 50), or (iii) Exploratory arm: DNA-personalised dietary advice via a self-guided app and wearable device (n = 46). The primary outcome was the difference in fasting plasma glucose (FPG) between the Control and Intervention arms after 6 weeks. 180 people were recruited, of whom 148 people were randomised, mean age of 59 years (SD = 11), 69% of whom were female. There was no significant difference in the FPG change between the Control and Intervention arms at 6 weeks (− 0.13 mmol/L (95% CI [− 0.37, 0.11]), p = 0.29), however, we found that a DNA-personalised dietary intervention led to a significant reduction of FPG at 26 weeks in the Intervention arm when compared to standard care (− 0.019 (SD = 0.008), p = 0.01), as did the Exploratory arm (− 0.021 (SD = 0.008), p = 0.006). HbA1c at 26 weeks was significantly reduced in the Intervention arm when compared to standard care (− 0.038 (SD = 0.018), p = 0.04). There was some evidence suggesting prevention of progression to T2DM across the groups that received a DNA-based intervention (p = 0.06). Personalisation of dietary advice based on DNA did not result in glucose changes within the first 6 weeks but was associated with significant reduction of FPG and HbA1c at 26 weeks when compared to standard care. The DNA-based diet was effective regardless of intervention type, though results should be interpreted with caution due to the low sample size. These findings suggest that DNA-based dietary guidance is an effective intervention compared to standard care, but there is still a minimum timeframe of adherence to the intervention before changes in clinical outcomes become apparent.

**Trial Registration:** www.clinicaltrials.gov.uk Ref: NCT03702465.

## Introduction

Obesity, type 2 diabetes mellitus (T2DM), and cardiovascular disease (including hypertension and dyslipidaemia), represent major global health challenges, and are associated with genetic, lifestyle, and environmental risk factors^1–10^. Lifestyle interventions including dietary modification can prevent progression to cardiometabolic disease in people at highest risk and can support optimal disease outcomes^11^.

Non-diabetic hyperglycaemia, which includes impaired fasting glucose (IFG), impaired glucose tolerance (IGT) and IFG with IGT, occurring prior to the onset of T2DM, is prevalent and provides an opportunity to prevent the progression of glucose intolerance. In 2017, an estimated 352.1 million people worldwide (7.3% of the global population), aged 20–79 years, lived with IGT, a number expected to rise to 531.6 million by year 2045^12^. Lifestyle interventions have been shown to effectively prevent or delay the progression to T2DM^13–15^.

General dietary and nutritional guidelines support evidence-based approaches to behaviour modification that are effective in a population, but may not provide specific or acceptable guidance for individuals^16,17^. Personalised dietary advice could, therefore, be an effective and acceptable alternative. An increasing evidence base supports the use of genetic information data for treatment personalisation in several conditions, such as cardiovascular disease^18–22^ and ischemic stroke^23^, and may be impactful for obesity and weight management^24–37^, improvements in dietary fat intake^30^, and even fasting blood glucose levels in obese participants^38^. Furthermore, a small number of studies has explored the gene-diet interaction in T2DM, providing initial evidence that T2DM risk could be modulated via DNA-personalisation of dietary interventions^39–42^.

There is existing literature regarding the benefits precision nutrition may have, both within the context of T2DM, but also in the broader context of health. One such study^16^, devised a machine-learning algorithm capable of predicting an individual’s postprandial glycaemic response to reported meals. The algorithm was utilised in a dietary intervention randomised controlled trial (RCT), the results of which demonstrated significant improvement in postprandial blood glucose. Another study on the same basis^43^, found that dietary guidance based on postprandial glucose predictions generated by a machine-learning algorithm was significantly better than a standard Mediterranean diet in patients monitored with constant glucose monitoring systems. Studies conducted using the PREDICT1 cohort, comprised of thousands of participants from both the UK and the US, demonstrated the potential value of personalised nutrition in predicting and managing postprandial food responses, and have successfully worked towards creating an extensive knowledge base to inform the development of personalised nutrition strategies^44–46^. It should also be mentioned that there have been many reviews of existing nutrigenomic work, such as studies which detail many nutrition-related genetic polymorphisms and their known functions^6,19,47,48^, as well as publications that have shown in clinical studies that the personalisation of dietary advice based on single nucleotide polymorphisms (SNPs) can have a significant effect on the management of populations that are at risk. For instance, Bo et al.^4^ investigated the role of SNP rs7903146 in *TCF7L2* (a SNP also investigated in the ASPIRE-DNA panel) on glucose values following a lifestyle intervention in a population of nondiabetic, dysmetabolic participants. Their findings revealed that, while lifestyle intervention led to metabolic improvement in all genetic subgroups, at the end of the trial, carriers of the risk allele presented with weight gain and developed first hyperglycemia and decreased insulin secretion, suggesting the need for preventive approaches personalised to the genotypic risk. Arkadianos et al.^38^, demonstrated that nutrigenetically tailored diets resulted in better compliance, longer-term body mass index (BMI) reduction and improvements in blood glucose levels. Horne et al.^30^, with the NOW RCT trial concluded that DNA-based weight management interventions can induce long-term significant improvements with regards to dietary fat intake compared to population-based interventions. Celis-Morales et al.^49^ demonstrated that the disclosure of information on *FTO* resulted in greater body weight and waist circumference reductions in *FTO* risk carriers. Furthermore, results from the Food4Me trial^50^, a multi-country, personalised dietary intervention RCT focused on changing intakes of unhealthy discretionary foods found that, when compared with generalised “healthy eating” advice, personalised nutrition advice was successful in significantly reducing participants’ intake of foods high in fat, added sugars, and salt^24,46–48^.

Additionally, there is already a wealth of information tying various SNPs to cardiometabolic and dietary biomarkers across the fields of nutrigenomics and personalised nutrition. For example, one study assessing the value of genetic risk scores in obesity found that participants carrying > 7 risk alleles had significantly higher BMI and body fat mass compared to participants with < 7 alleles^24^. The same authors went on to conduct the NUGENOB trial^51^, that demonstrated an interaction between rs1440581 and changes in glucose metabolism during weight loss, in line with dietary intakes of fats and carbohydrates. This work was further supported by an extensive GWAS study of 180,834 individuals with T2DM (and 1,159,055 controls), of multiple ancestries (48.9% non-European), that identified 237 associated genetic loci and laid the foundations for functional investigations^52^. In more general work, there have been studies linking the phenotypic expressions of T2DM and obesity to underlying genetic mechanisms such as DNA methylation^53^, which continue to develop the framework for understanding the genetic basis of chronic non-communicable diseases like T2DM.

People with one parent with T2DM have a 40% risk of developing T2DM within their lifetime, a number that increases to 70% if both parents have T2DM^54^. However, Diabetes Prevention Programmes (DPPs) with face-to-face contact can be expensive and labour-intensive, with multiple personal contacts required^55^. The ASPIRE-DNA study was an open label RCT that aimed to assess the use of genetic data to inform personalised nutritional recommendations in people with non-diabetic hyperglycaemia, and to explore the impact of personalised nutrition on glucose metabolism, non-glucose metabolism, anthropometry, and lifestyle.

In addition to the traditional approach of healthcare professional encounters, the ASPIRE-DNA trial investigated the effect of a self-guided, smartphone app and accompanying wearable technology for the delivery of DNA-personalised information that could be constantly accessed by the user, at their discretion.
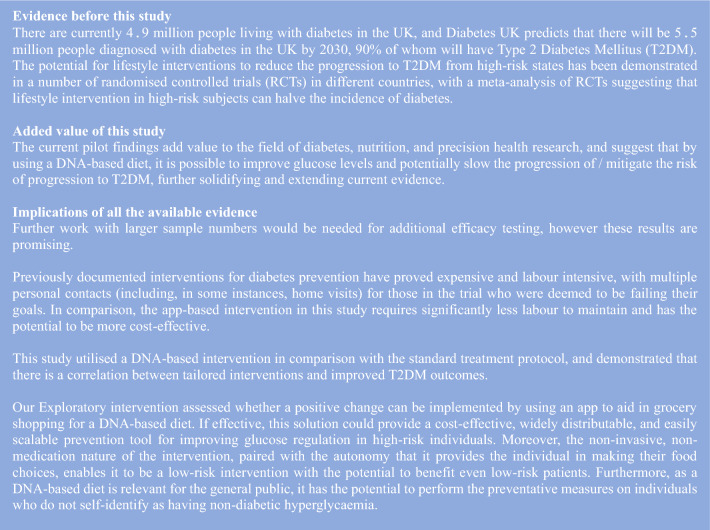


## Methods

### Study design and participants

**Figure 1:**
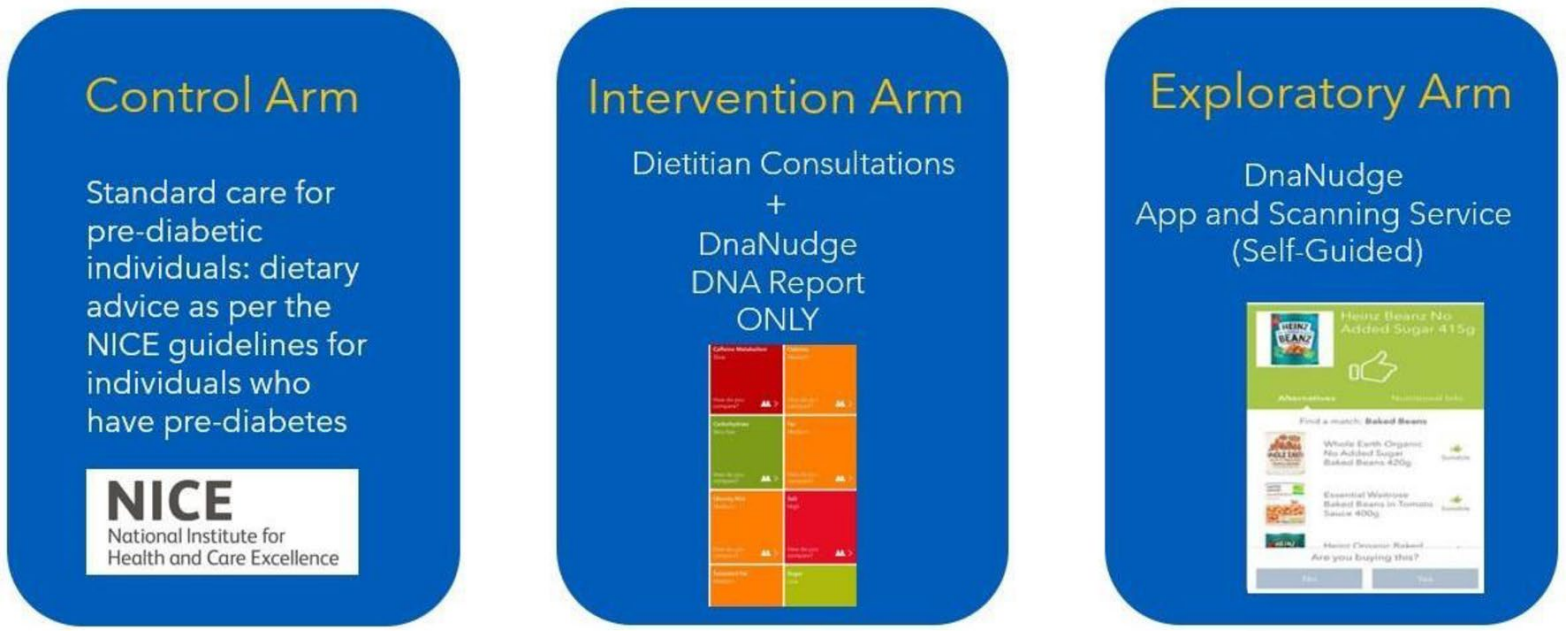
Breakdown of each study arm.

ASPIRE-DNA was an open label pilot study with a trial design involving two parallel arms (Intervention versus active Control), and an Exploratory arm. 148 people were randomised, mean age of 59 years (SD = 11), 69% of whom were female. Participants with non-diabetic hyperglycaemia (HbA1c: 42–47 mmol/mol (6–6.4%)) were randomised 1:1:1 to either (i) the Control arm, (ii) the Intervention arm, or (iii) the Exploratory arm, as depicted in Fig. 1.

The Control arm received general healthy eating dietary advice according to the NICE guidelines via regular consultations with a Dietitian; the consultation followed the standard NHS procedures and structure, including setting dietary goals in line with the NICE guidelines.

**Figure 2:**
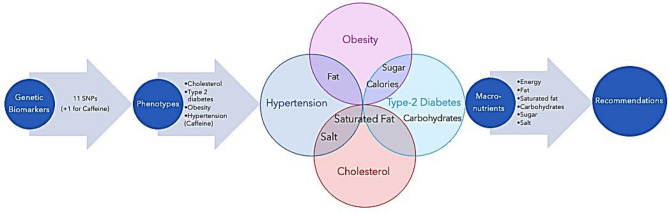
Overview of the DNA-based product recommendations architecture.

The Intervention arm participants had a DNA test that included genetic biomarkers associated with the development of obesity, hypertension, T2DM, and blood cholesterol (biomarkers listed in Table S2 in the Electronic Supplementary Material; ESM). Multiple variants were used per phenotype; each SNP was assigned a weight factor, and a genetic risk score was generated per phenotype. The participants were provided with a genetic report that included information about their long-term health sensitivity to fat, saturated fat, carbohydrate, sugar, salt, and calories, (please refer to the ESM, Fig. S1 for an example of a genetic report) alongside with regular consultations with a Dietitian to discuss dietary goals and progress as they related to each individual’s macronutrient profile (for more information, please see ESM). Figure 2 summarises the mapping between the genetic risk for the given chronic conditions (i.e. obesity, T2DM, hypertension, and cholesterol) and the macronutrients (i.e. energy, fat, saturated fat, carbohydrates, sugar, and salt). The chronic disease-macronutrient associations have been derived from established guidelines and dietetics studies (e.g., hypertension has been mapped to salt, saturated fat, and fat in accordance with international dietary guidelines, such as the DASH eating plan). For instance, if an individual carried high risk copies for the SNPs associated with hypertension (i.e. *AGT* rs699 and *CSK*rs1378942), the Dietitian would advise them accordingly about salt consumption.

The Exploratory arm received DNA-personalised dietary advice via a genetic report (the same format as the Intervention arm) and an accompanying self-guided app and wearable device that could scan food barcodes. The Exploratory group did not have consultations with a Dietitian, and their intervention was entirely self-guided. Participant’s genetic information was used to customise food and drink product recommendations in order to find healthier product options when they went shopping. For products not recommended, the app suggested other products in the same product category that might suit the individual better.

Randomisation was completed using stratified blocked randomisation, each block having a maximum of 60 participants, utilising an online secure randomisation tool called “Sealed Envelope”. Randomisations and participant enrolments were conducted by the Research Nurse.

Where they occurred, the main reasons for any withdrawals of participants from the trial were either that the participant went into T2DM, or that they were lost to follow up. There were sporadic instances of withdrawals due to other factors, such as other health conditions not related to the trial, or self-withdrawal at the request of the participant. When a participant was withdrawn, they ceased the intervention, no further visits were conducted, but where possible the Research Nurse liaised with the participant to document the reasons for withdrawal and to answer any questions, as well as passing on any relevant information to their GP (where consent to do so was provided).

Participants were recruited across the course of 2019–2022 and ceased when the 180th participant completed their Baseline visit. The final participant completed their follow-up period in October 2022, at which point the trial was officially ended. 148 participants were officially randomised to an intervention, as some participants were withdrawn at or after their baseline visit.

The primary objective was to compare differences in the impact of a DNA-based diet and standard care in improving glucose regulation in individuals with non-diabetic hyperglycaemia. The primary outcome being the difference in 0 min glucose on 75 g oral glucose tolerance test (OGTT)—reported as Fasting Plasma Glucose (FPG)—between the Control arm and the Intervention arm at 6 weeks.

The secondary objective was to assess the ability of a DNA-based diet to improve the macro- and micro-nutrient profile of participants with non-diabetic hyperglycaemia; to assess the impact of a DNA-based diet on clinical markers including cholesterol, body composition, and BP, as they relate to diabetes risk, and, where applicable; to assess changes in anthropometric measurements as a result of following a DNA-based diet, where they relate to diabetic risk reduction. The secondary outcomes were 0 min glucose on 75 g oral glucose tolerance test (reported as FPG), and 120 min glucose on 75 g oral glucose tolerance test (reported as 2 h plasma glucose; 2 h-PG) (please see protocol for the full list). All secondary outcomes were measured at 6, 12, 26 weeks (with the exception of HbA1c, which was only measured at 12 and 26 weeks).

The exploratory objectives were to assess the impact of providing DNA-based dietary guidelines via the DnaNudge app on improving glucose regulation in individuals with non-diabetic hyperglycaemia. To assess the impact of providing DNA-based dietary recommendations via the DnaNudge app on the secondary outcomes, and to explore the utility of an app as a delivery mechanism for DNA-based dietary advice.

Participants were screened prior to entry into the study. Potential participants were invited to the clinical research facility (CRF) where they were informed on the consent procedure and given the opportunity for questions. Following consent and verification that they were eligible according to the inclusion and exclusion criteria, participants underwent a glucose regulation test by providing a finger prick blood sample that was analysed by a lab-based analyser, to measure HbA1c. The HbA1c test results were interpreted according to the WHO criteria, with eligibility determined at screening by HbA1c criteria (6.0–6.4%) only. Ongoing eligibility for the study was determined by an OGTT and laboratory HbA1c. Participants with IFG, IGT, and HbA1c that were within the non-diabetic hyperglycaemic range were randomised.

Participants were reviewed after 6 and 12 weeks with repeat baseline measures. Those no longer eligible were withdrawn from the study. Participants were also asked to complete a Food Frequency Questionnaire (FFQ) before their initial dietitian consultation and at the 6-, 12-, and 26-week follow up clinical visits. Participants in the Control and Intervention groups also completed a 24-h Food Recall at each follow-up phone call with the Dietitian (Visits 5, 7, 9, 11). Further information on eligibility criteria is provided in the ESM, and a full schedule can be found in the study protocol.

This research has been approved by the North of Scotland Research Ethics Service (Ref: 18/NS/0093), and informed consent was given by all participants prior to enrolment. All research activities were performed at the Imperial Clinical Research Facility at Hammersmith Hospital, London. All methods were performed in accordance with the relevant guidelines and regulations. Further details on the randomisation process, time periods of recruitment and follow up, and sample size estimation from the power calculation are provided in the trial protocol, which can be accessed at clinicaltrials.gov (Ref: NCT03702465, first registration 11/10/2018).

DNA analysis was performed by standard lab polymerase chain reaction (PCR) testing (using a Roche, LightCycler® 96 Instrument), and consisted of DNA extraction from saliva samples, followed by subsequent DNA amplification using DnaNudge allele-specific primers for the detection of SNPs associated with nutrition-related health conditions. SNPs associated with genetic propensity (please refer to ESM Table S2) for the development of obesity, T2DM, hypertension, and high cholesterol were selected via a rigorous process that assessed published studies such as Genome Wide Association Studies (GWAS) (including the manually curated GWAS Catalogue), scientific research papers further validating GWAS findings, clinical trials, and meta-analyses, all of which were unbiased analyses for the discovery of genomic variants associated with the phenotypes of interest. The SNPs assessed for are as follows: rs10811661, rs1367117, rs1378942, rs1558902, rs2479409, rs4420638, rs6065906, rs6567160, rs699, rs762551, rs7903146, rs8050136.

### Statistical methods

The primary outcome of the analysis was to compare the difference in fasting glucose on 75 g OGTT between the Control arm and the Intervention arm at 6 weeks. Secondary outcomes were cross-arm and within-arm differences (compared to baseline measurements) between the Control arm, Intervention arm, and the Exploratory arm, in respect of fasting glucose, 120 min glucose on 75 g OGTT, HbA1c, weight, BMI, lean mass, fat mass, waist circumference, total cholesterol, fasting triglycerides, LDL cholesterol, HDL cholesterol, Homeostasis Model Assessment Insulin Resistance test (HOMA-IR) and Homeostasis Model Assessment β-cell function test (HOMA-B), 120 min c-peptide following 75 g OGTT, systolic BP, diastolic BP, food frequency (by FFQ and 24 h Food Recall), energy intake, carbohydrate intake, fat intake, saturated fat intake, salt intake, vitamin D, vitamin B6, and vitamin B12. Both primary and secondary outcomes were measured at 6-, 12-, and 26-weeks.

For the primary outcome the intention to treat (ITT) analysis was used. Week 6 FPG was compared between groups using ANCOVA with adjustment for age, sex, and baseline outcomes^56^. The sample size was computed to detect differences between arms in primary outcomes of − 0.5 mmol/L with a power of 80% and a Type 1 error^57^ set at 0.05. It was calculated that with 60 participants in each group, and assuming that the average FPG changes from baseline to week 6 would be approximately − 0.6 mmol/L in the intervention arm and − 0.1 mmol/L in the control arm, that the baseline standard deviation (SD) of FPG would be 0.7, the post-intervention SD would be 0.6, and that the correlation between baseline and post-intervention measurement range would be between 0.6 and 0.4, the analysis would have a power greater than 0.9 to detect a difference between both arms.

Repeated measures analyses were also performed with linear mixed model or generalised additive mixed model (GAMM) to assess the changes in the outcomes over the follow-up period^58^; mixed models were fitted to all available data. GAMM was used to account for non-linear effect of covariates. Covariates were centred in the analysis and some variables were transformed using the Box-Cox transformation to achieve a distribution closer to normality^59^. For variables showing departure from normality, the non-parametric Mann Whitney Wilcoxon rank sum test (for two arms) or the Kruskal and Wallis test (used to compare the three arms), and the rank-based regression of the package Rfit, or the robust linear regression lmrob from the R package robust base (for multivariate regression) were used. Missing data were imputed for participants not lost-to-follow up under the assumption that missingness was at random.

Sensitivity analysis was conducted to check for important differences that would suggest a significant influence of variable distribution on the parameters estimates. This analysis was comprised of a comparison of the results using a linear standard regression model, a robust linear regression model, and a non-parametric linear regression model. No significant differences were found.

Participants were considered to have progressed to T2DM at a given visit if they had an FPG ≥ 6.9 mmol/L or 2 h-PG > 11 mmol/L at that visit. The overall progression to T2DM was defined as a progression to diabetes at any visit during follow-up. As the time to progression was right censored because some participants did not complete the 26 weeks follow-up, the non-parametric Kaplan Meyer estimator was used to estimate the probability of progression to T2DM.

The analyses were performed with R version 4.2.2 and used a significance level α of 0.05. Because of potential type I error from multiple comparisons, findings from secondary analyses should be considered exploratory.

## Results

Baseline demographics were similar across arms of the study (the full socio-demographic and baseline biological characteristics are depicted at Table 1; primary and secondary outcomes are summarised in Table 2).

**Table 1:** Socio-demographic and baseline biological characteristics of study participants.

	Global	Control	Intervention	Exploratory
	n = 147*	n = 51	n = 50	n = 46
Socio-demographic characteristics				
Sex, N (%)				
Female	101 (69)	35 (69)	35 (70)	31 (67)
Male	46 (31)	16 (31)	15 (30)	15 (33)
Age (year)				
Median (interquartile range)	60 (50–67)	57 (46–65)	60 (50–67)	60 (55–68)
Ethnicity, N (%)				
White	28 (21)	12 (27)	9 (19)	7 (16)
Black	23 (17)	8 (18)	8 (17)	7 (16)
Asian	68 (50)	17 (39)	28 (58)	23 (52)
Other	17 (12)	7 (16)	3 (6)	7 (16)
Baseline biological characteristics, mean (SD)				
HbA1c (mmol/mol)	44.1 (2.3)	44 (2.57)	44.1 (2.1)	44.1 (2.1)
FPG (mmol/L)	5.32 (0.59)	5.12 (0.48)	5.38 (0.61)	5.49 (0.61)
2 h PG (mmol/L)	6.95 (1.86)	6.97 (2.01)	6.99 (1.84)	6.87 (1.74)
C-peptide (pmol/L)*	2038 (1613)	1894 (1556)	2128 (1668)	2089 (1637)
Insulin (mUI/L)*	33.9 (43.62)	29.83 (35.71)	37.78 (52.44)	33.83 (40.69)
HOMA-IR	3.8 (4.17	3.5 (3.79)	3.91 (4.45)	4 (4.32)
HOMA-B	205 (172)	208 (180)	208 (180)	198 (159)
Total cholesterol (mmol/L)	5.22 (1.12)	5.24 (1.06)	5.22 (1.01)	5.21 (1.31)
HDL-cholesterol (mmol/L)	1.41 (0.35)	1.4 (0.41)	1.44 (0.35)	1.38 (0.28)
LDL-cholesterol (mmol/L)*	3.25 (1.02)	3.34 (0.96)	3.19 (0.99)	3.23 (1.14)
Triglycerides (mmol/L)	1.29 (0.61)	1.25 (0.56)	1.3 (0.63)	1.32 (0.65)
Weight (kg)	83.7 (19.8)	82.6 (19.5)	84.6 (18.9)	83.7 (21.6)
BMI (kg/m^2^)	30.4 (6.9)	30.3 (6.5)	30.3 (6.5)	30.4 (8.3)
Fat mass (kg)	29.6 (13.7)	30 (13)	30.3 (12.3)	28.4 (15.9)
Lean mass (kg)*	51.3 (10.9)	50.1 (10.5)	51.4 (12)	52.5 (10.1)
Waist circumference (cm)	101.7 (15.6)	101.4 (15.8)	102.4 (14.8)	101.2 (16.7)
Systolic BP (mmHg)	125.8 (13.5)	124.1 (13.1)	102.4 (14.8)	101.2 (16.7)
Diastolic BP (mmHg)	71.6 (9.8)	70.8 (9.1)	71.3 (9.7)	72.9 (10.8)

* 148 participants were randomised, however one participant did not have Baseline data; n = 147.

**Table 2:** Primary and secondary outcomes at weeks 6, 12, and 26.

Variable	Reference (control group; mean (SD))	Difference between control and intervention groups; (mean (SD))	p-value	Difference between control and exploratory groups (mean (SD))	p-value
Week 6					
FPG (mmol/L)	5.31 (0.72)	− 0.13 (0.12)	0.28	− 0.22 (0.13)	0.09
FPG Change from Baseline over time (mmol/L)	5.12 (0.48)	− 0.15 (0.12)	0.22	− 0.23 (0.13)	0.07
2 h PG (mmol/L)	7.38 (2.21)	− 0.99 (0.354)	0.01	− 0.51 (0.384)	0.18
Systolic BP (mmHg)	123 (16)	− 1.8 (2.9)	0.54	1.82 (3.03)	0.55
Diastolic BP (mmHg)	71 (10)	− 2.74 (1.8)	0.13	0.06 (1.9)	0.98
Total cholesterol (mmol/L)	5.05 (1.13)	− 0.08 (0.13)	0.55	0.25 (0.14)	0.07
Triglycerides (mmol/L)	1.25 (0.66)	− 0.07 (0.07)	0.38	0.06 (0.08)	0.42
HDL cholesterol (mmol/L)	1.3 (0.29)	0.04 (0.04)	0.33	0.04 (0.04)	0.32
Weight (kg)	81 (20)	− 0.93 (0.79)	0.24	1.4 (0.81)	0.09
BMI (kg/m^2^)	30 (7)	− 0.31 (0.28)	0.26	0.41 (0.29)	0.16
Fat mass (kg)	29 (13)	− 1.12 (0.59)	0.06	1.01 (0.62)	0.11
Waist circumference (cm)	101 (17)	− 1.59 (0.87)	0.07	0.03 (0.9)	0.98
HOMA-IR	5.1 (5.19)	− 0.91 (0.8)	0.23	0.03 (0.83)	0.85
HOMA-B	256 (215)	− 25.24 (32.33)	0.38	17.74 (334.45)	0.51
12 weeks					
FPG (mmol/L)	5.16 (0.66)	0.07 (0.12)	0.54	− 0.09 (0.12)	0.45
FPG Change from Baseline over time (mmol/L)	5.12 (0.48)	0.06 (0.12)	0.61	− 0.1 (0.12)	0.38
HbA1c (mmol/mol)	43.55 (2.92)	− 0.5 (0.46)	0.28	− 0.53 (0.45)	0.24
2 h-PG (mmol/L)	6.79 (2.3)	− 0.41 (0.36)	0.3	0.25 (0.35)	0.44
Systolic BP (mmHg)	119 (10.5)	− 0.83 (2.86)	0.77	3.06 (2.81)	0.28
Diastolic BP (mmHg)	69.8 (8.8)	− 0.88 (2.11)	0.68	− 0.34 (2.08)	0.87
Total cholesterol (mmol/L)	5.04 (1.2)	0.031 (0.17)	0.94	0.26 (0.17)	0.09
Triglycerides (mmol/L)	1.19 (0.6)	0.02 (0.08)	0.78	0.1 (0.08)	0.29
HDL cholesterol (mmol/L)	1.36 (0.31)	0.06 (0.05)	0.32	0.05 (0.05)	0.27
Weight (kg)	78.1 (18.3)	− 1.54 (0.93)	0.1	1.33 (0.91)	0.15
BMI (kg/m^2^)	28.5 (6.3)	− 0.48 (0.34)	0.16	0.43 (0.33)	0.21
Fat mass (kg)	26.6 (12.5)	− 1.91 (0.76)	0.01	1.39 (0.75)	0.07
Waist circumference (cm)	98.2 (15.9)	− 2.31 (1.24)	0.07	1.19 (1.21)	0.33
HOMA-IR	4.6 (5.14)	0.15 (0.914)	0.91	− 0.45 (0.91)	0.71
HOMA-B	258.5 (238.6)	− 1.55 (35.33)	0.94	− 14.35 (35.15)	0.72
26 weeks					
FPG (mmol/L)	5.45 (1.25)	− 0.17 (0.12)	0.14	− 0.19 (0.12)	0.12
FPG Change from Baseline over time (mmol/L)	5.12 (0.48)	− 0.21 (0.12)	0.09	− 0.21 (0.13)	0.1
HbA1c (mmol/mol)	43.68 (5.96)	− 1.77 (0.87)	0.05	− 0.9 (0.88)	0.31
2 h-PG (mmol/L)	6.34 (2.66)	0.13 (0.4)	0.72	0.014 (0.42)	0.86
Systolic BP (mmHg)	126.79 (11.45)	− 7.8 (3.81)	0.04	− 1.84 (3.83)	0.63
Diastolic BP (mmHg)	72.21 (8.81)	− 4.1 (2.37)	0.09	− 0.4 (2.41)	0.87
Total cholesterol (mmol/L)	5.23 (1.19)	− 0.19 (0.17)	0.24	− 0.07 (0.17)	0.68
Triglycerides (mmol/L)	1.2 (0.61)	− 0.03 (0.1)	0.78	0.02 (0.1)	0.88
HDL cholesterol (mmol/L)	1.41 (0.43)	0.07 (0.04)	0.13	0.02 (0.04)	0.57
Weight (kg)	78.72 (18.94)	− 0.7 (1.24)	0.58	1.26 (1.24)	0.31
BMI (kg/m^2^)	28.72 (6.58)	− 0.16 (0.45)	0.73	0.42 (0.45)	0.36
Fat mass (kg)	27.52 (13)	− 1.01 (1.05)	0.34	1.79 (1.05)	0.09
Waist circumference (cm)	97.98 (16.35)	− 1.95 (1.52)	0.2	1.74 (1.52)	0.26
HOMA-IR	4.11 (4.98)	− 0.008 (0.12)	0.47	0.29 (0.31)	0.18
HOMA-B	203.9 (172.9)	19.7 (20.43)	0.17	30.62 (30.9)	0.16

The difference between arms has been estimated using the ANCOVA method, adjusted for baseline results.

**Figure 3:**
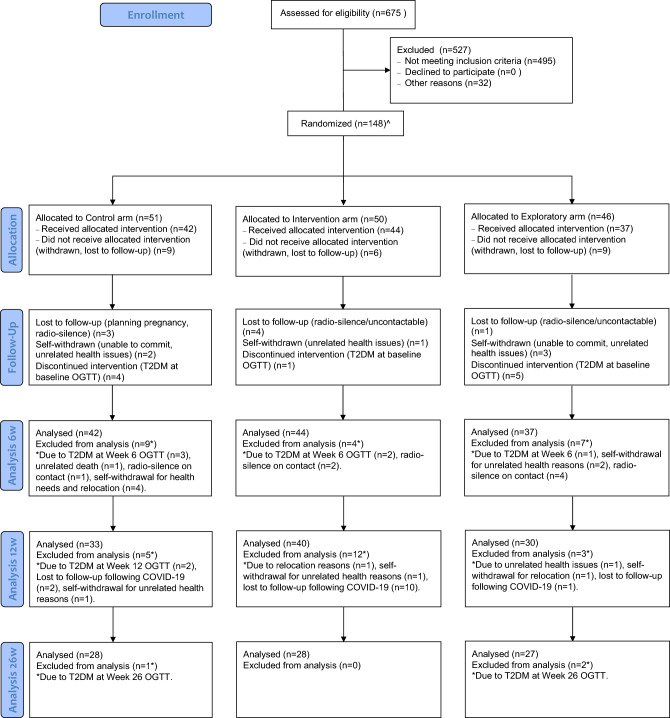
CONSORT diagram of cohort structure across the course of the trial. ^Of the 148 randomised, 1 participant did not have Baseline data.

A total of 148 participants have been randomised, of which 123 completed the week 6 visit (Control arm: 42, Intervention arm: 44, Exploratory arm: 37), 100 completed the week 12 visit (Control arm: 34, Intervention arm: 32, Exploratory arm: 34), and 83 completed the week 26 visit (Control arm: 28, Intervention arm: 28, Exploratory arm: 27). Figure 3 includes the cohort structure diagram, detailing the randomisation into groups^60^.

After adjusting for baseline FPG, there was no significant difference in the primary outcome of fasting glucose between the Control and Intervention arms at week 6 (− 0.13 mmol/L (95% CI [− 0.37, 0.11]), p = 0.28). At the same timepoint, the Exploratory arm showed some reduction of FPG (− 0.22 mmol/L (95% CI [− 0.47, 0.04]), p = 0.09), although not statistically significant.

Sub-group analysis by sex for the primary outcome, and adjusted with age and baseline FPG showed a modest change in the Intervention group in males (− 0.46 (SD = 0.23), p = 0.06), and no change in female participants (0.11 (SD = 0.12), p = 0.38), and no changes in the Exploratory group in either males (− 0.31 (SD = 0.25), p = 0.23) or females (− 0.13 (SD = 0.13), p = 0.31).

**Figure 4:**
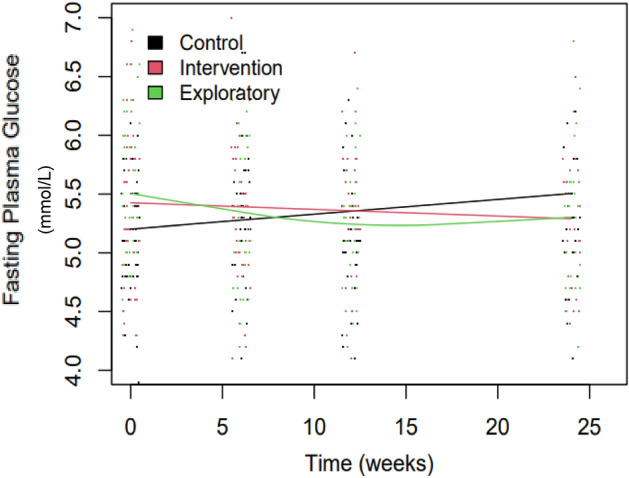
Changes in FPG over time. Dots represent observed values. Lines are the fitted regression lines using a generalised additive mixed-effect model (GAMM). Mixed models were fitted to all available data.

Regarding secondary outcomes, lower 2 h PG was observed in the Intervention arm at week 6 (− 1.02 mmol/L (95% CI [− 1.74, − 0.30]), p = 0.01). Across the overall follow up period of 26 weeks, longitudinal analyses demonstrated a greater decrease in FPG (Fig. 4) in both the Intervention and Exploratory arms when compared with the Control arm (− 0.019 (SD = 0.008), p = 0.01) and (0.021 (SD = 0.008), p = 0.006) respectively), (please refer to Table 3 for further details, including the results with the robust version of lme4 package estimates), while a greater decline in HbA1c (− 0.038 (SD = 0.018), p = 0.04) was observed in the Intervention arm. There was no significant change in HbA1c in the Exploratory arm (− 0.025 (0.031), p = 0.4) (Table 3). Longitudinal analyses showed a significant reduction in systolic BP (− 7.8 (SD = 3.81), p = 0.04), observed in the Intervention arm only. The Intervention arm also showed reduced overall weight, and reductions in BMI, fat mass, and waist circumference at all weeks, but these results were not statistically significant.

**Table 3:** Changes in FPG and HbA1c outcomes over 26 weeks; difference between control, Intervention, and exploratory arms. Differences are estimated with linear mixed effects models. FPG is measured in mmol/L, HbA1c is measured in mmol/mol.

	Intervention		Exploratory	
	Diff (sd)	p-value	Diff (sd)	p-value
FPG	− 0.019 (0.008)	0.01	− 0.021 (0.008)	0.006
HbA1c	− 0.038 (0.018)	0.04	− 0.025 (0.031)	0.4

With the robust version of the lme4 package estimates are				
	Diff (sd)	p-value	Diff (sd)	p-value
FPG	− 0.011 (0.005)	0.03	− 0.014 (0.005)	0.008
HbA1c	− 0.038 (0.018)	0.04	− 0.003 (0.018)	0.85

Differences are estimated with linear mixed effects models. FPG is measured in mmol/L, HbA1c is measured in mmol/mol.

There was a significant reduction in carbohydrate intake over 26 weeks in both the Intervention arm ((− 0.03 (95% CI [− 0.050, − 0.001]), p = 0.01), and the Exploratory arm (− 0.03 (95% CI [− 0.055, − 0.008]), p = 0.01) respectively), as shown in Table 5.

**Figure 5:**
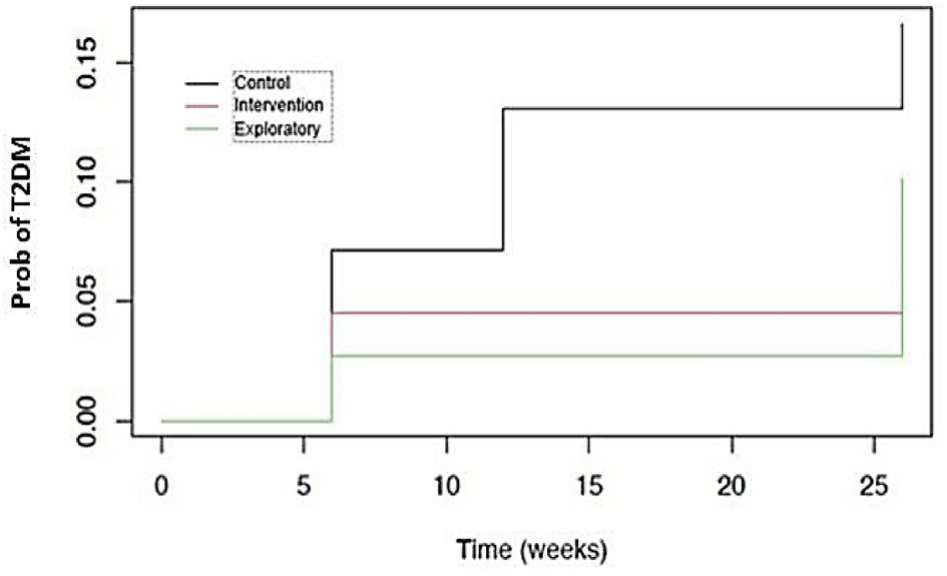
Participant probability of progression to T2DM.

There was some evidence to suggest the progression to T2DM was prevented (Fig. 5). The estimated cumulative hazards of progression by week 26 were; Control arm: 0.16 (95% CI [0.03, 0.27]); Intervention arm: 0.05 (95% CI [0, 0.11]); and Exploratory arm: 0.1 (95% CI [0, 0.20]), p = 0.06 (Table 4).

**Table 4:** Progression of participants to T2DM; Number of participants (proportion of group).

	Week 6		Week 12		Week 26	
	No	Yes	No	Yes	No	Yes
Control	39 (92.9)	3 (7.1)	32 (94.1)	2 (5.9)	27 (96.4)	1 (3.6)
Intervention	42 (95.5)	2 (4.5)	32 (100)	0 (0)	28 (100)	0 (0)
Exploratory	36 (97.3)	1 (2.7)	34 (100)	0 (0)	25 (92.6)	2 (7.4)

## Discussion

A diagnosis of non-diabetic hyperglycaemia, or T2DM, can be a life-changing event associated with significant health-related challenges. In the ASPIRE-DNA study we sought to investigate whether dietary advice tailored to individual DNA information would be superior to that of standard care, and whether the impact can be realised with a digital implementation.

While the primary outcome at 6 weeks did not show a statistically significant change, (− 0.13 mmol/L (95% CI [− 0.37, 0.11]), p = 0.28), the ASPIRE-DNA trial results suggest that a DNA-based intervention may yield positive clinical results by significantly decreasing FPG and HbA1c, and by reducing the risk of progression to T2DM over 26 weeks. Our findings demonstrate a significant reduction of FPG at 26 weeks in both the Intervention arm (− 0.019 (SD = 0.008), p = 0.01), and the Exploratory arm (− 0.021 (SD = 0.008), p = 0.006) when compared to the Control arm. HbA1c at 26 weeks was significantly reduced in the Intervention arm when compared to standard care (− 0.038 (SD = 0.018), p = 0.04). There was some evidence suggesting prevention of progression to T2DM across the groups that received a DNA-based intervention (p = 0.06). Importantly for implementation at scale, this was true regardless of the modality of the delivery of the DNA-based dietary advice (1-2-1 Dietitian sessions versus self-guided use of an app and a wearable device). It is well known that weight loss reduces the risk of developing T2DM^61–65^, which is supported by the findings of randomised control trials in samples with non-diabetic hyperglycaemia, demonstrating that the onset of T2DM can be prevented or delayed through behavioural interventions that promote weight loss, increase physical activity, and improve the quality of nutritional intake^3,66,67^. Interestingly, in the present study, participants in the Intervention and Exploratory arms showed improved FPG without observable changes in weight loss, possibly suggesting that personalised dietary guidance could be an important component in improving glucose regulation. Though clinical research into personalised nutrition and T2DM is still developing, there is already substantial evidence supporting the practical and clinical value of personalised approaches of various modalities^68,69^.

The FFQs showed cross sectional differences in the respective food intake in the Intervention and Exploratory arms. Despite differences in food intake, both arms still had reduced glucose measures after 26 weeks (Table 5). This could suggest that the intervention being DNA-based is key, rather than the mode of intervention.

**Table 5:** Change in food intake over 26 weeks and differences between arms, compared to the control group, after Box–Cox transformation when compared with the control group (analyses adjusted for age, sex, and baseline values).

	Difference between control arm and intervention arm	Difference between control arm and exploratory arm
Energy	− 0.01 (0.01), p = 0.444	0 (0.01), p = 0.97
Salt	0 (0.01), p = 0.77	0.01 (0.01), p = 0.39
Carbohydrate	− 0.03 (0.01), p = 0.04	− 0.03 (0.01), p = 0.01
Fat	0 (0.01), p = 0.74	0.01(0.01), p = 0.55
Saturated fat	0 (0.01), p = 0.97	0.01 (0.01), p = 0.53

These results are in line with the well-established field of precision nutrition and support the increasing evidence that a DNA-personalised intervention could be a valuable tool with potential for far-reaching positive impacts in diabetes prevention and management^17,70–72^. The preserved impact in the digital implementation is particularly important for scalability as a population level intervention and has value in the realm of modern digital interventions, an evolving area of healthcare described by numerous studies and perspectives papers^73–76^.

As an adjunct to existing pathways, there is a need for exploration with future studies assessing the impact of a DNA-based diet, powered for diabetes prevention, and assessing alternative cardiometabolic outcomes, including cardiovascular outcomes, diabetes management, and obesity management, in conjunction with DNA-personalised dietary interventions. Finally, as the existing literature consistently suggests, we agree that further research and investigations into factors surrounding patient and participant engagement with digital health interventions is needed to efficiently implement these tools in healthcare at scale^73^.

This study was registered at clinicaltrials.gov, under reference NCT03702465, and all activities were performed at the Imperial Clinical Research Facility at Hammersmith Hospital, London.

### Study strengths and limitations

This study assessed the impact of a novel intervention in a high-risk population.

The Intervention arm had more than double the number of Asian participants compared to the Control arm. This difference occurred by chance, and while ethnic differences in the progression of non-diabetic hyperglycaemia have been reported^66,77,78^, the short duration of the study minimised the influence of ethnicity on the primary outcome. Additionally, it should be noted that any genetic risk factors for T2DM could have limited effect in comparison to other biological or socioeconomic vulnerabilities, and disparities in access to healthcare tied to race and ethnicity^79,80^.

The ASPIRE-DNA study was affected by the COVID-19 pandemic, with many participants lost to follow up during lockdown restrictions and across the suspension of the study. The study was able to recover with new recruitment paths post-lockdown.

Unfortunately, due to the natural risks of progression to T2DM in individuals with impaired glucose metabolism, a number of participants had to be withdrawn, in addition to high numbers of participants lost to follow up. As such, this is not a fully powered study, as the final sample sizes for the analysis fell below the levels required by the original power calculation. As such, the authors recommend a degree of caution in interpreting these results, due to the smaller sample sizes. This also, unfortunately, may mean that these results have lower generalisability than expected, as well as lower statistical power than planned, and would warrant further validation studies to confirm the results in a larger sample.

In terms of analyses, as there was no adjustment rule for multiple testing for the secondary outcomes, we would recommend the results for the secondary outcomes should be interpreted as exploratory only. Further validation studies are needed to confirm these results on a larger scale and in more detail. Moreover, the effect of the herein described DNA-based dietary intervention could be explored in other clinical conditions where glucose management is impaired, for instance in individuals with T2DM (insulin independent or dependent), or individuals with Type 1 Diabetes.

## Conclusions

This trial is an early investigation of the effects of DNA-based dietary guidance on people with non-diabetic hyperglycaemia, compared with standard care over a period of 26 weeks. The results suggest that DNA-based dietary guidance may reduce FPG levels and potentially delay progression to T2DM for high-risk groups. These are a promising indication of the benefits of using a DNA-personalised approach to diabetes prevention, and warrant further exploration and investigation.

### Supplementary Information

Supplementary Information.

## Data Availability

The datasets generated during and/or analysed during the current study are available in an anonymised format from the corresponding author on reasonable request. Related documents (e.g. statistical analysis plan, informed consent form) are available on reasonable request with a signed data access agreement.

## Code availability

All data were analysed using R. The scrips are available on reasonable request.
