# Supplementary material for: Assessment of the impact of a personalised nutrition intervention in impaired glucose regulation over 26 weeks: a randomised controlled trial
Source: Sci Rep. 2024 Mar 5;14:5428. doi: 10.1038/s41598-024-55105-6 (PMC10914757; doi:10.1038/s41598-024-55105-6)
Supplement: Supplementary file 1 — Supplementary Information. [file 41598_2024_55105_MOESM1_ESM.docx]

**Electronic Supplementary Material**

# Trial eligibility criteria

Inclusion criteria:

- Adults over 18 years of age.
- Impaired glucose regulation including IFG and IGT by fasting glucose, OGTT or HbA1c criteria.
- Access to smartphone with an operating system of iOS 8.0 or above, or Android 4.0 or above.

Exclusion criteria:

- Diabetes
- Pregnant or planning pregnancy
- Breastfeeding
- Enrolled in other clinical trials
- Have active malignancy or under investigation for malignancy.
- Severe visual impairment
- Reduced manual dexterity
- Use of psychiatric, anti-diabetic, and/or weight loss medication, and/or oral steroids.
- Bariatric surgery
- History of illnesses that could interfere with the interpretation of the study results (e.g. HIV, Cushing syndrome, chronic kidney disease, chronic liver disease, hyperthyroidism, hereditary fructose intolerance, alcohol or substance abuse).
- Unable to participate due to other factors, as assessed by the Chief Investigator.

# Dietary Intervention

Both the Control (NICE guidelines) and Intervention (DNA-based dietary guidelines) arms were provided with their respective dietary interventions via a consultation with a Dietitian, whereby they were given a hard-copy of the relevant guidelines. The Control group received the standard NHS NICE guidelines for non-diabetic hyperglycaemia in both hard copy format, and verbally during their consultation with the Dietitian; the consultation followed the standard NHS procedures and structure, including setting dietary goals in line with the NICE guidelines. The Intervention group received their personalised DNA-based guidelines in both hard copy format, and verbally during their consultation with the Dietitian; the consultation included an explanatory breakdown of their DNA-based guidelines, what each item in the report referred to, and guidance and goals specific to their personalised guidelines.

Participants in both arms were contacted by the Dietitian 2 weeks following the initial consultation to answer any questions they may have. The purpose of the phone-calls was to provide clarity and therefore no additional dietary guidelines were provided.

## DNA-based Dietary Guidelines: Overview

The DNA-based dietary guidelines are derived from the technology and algorithms of DnaNudge Ltd. A cheek swab is used to take saliva from the participant, and a genetic test is performed using a Roche, LightCycler® 96 Instrument on a targeted number of SNPs that have been associated with risk for T2DM, obesity, cholesterol and hypertension risk (this forms the nutrition panel). The result of the genetic test is then incorporated, via the proprietary algorithms of DnaNudge Ltd, with national dietary guidelines for macronutrients, and the dietary advice of a Dietitian. Dietitians within DnaNudge Ltd have adjusted the DNA-based guidelines to be tailored to the macronutrient advice they would provide to someone with a given risk profile for the aforementioned chronic conditions. The resulting DNA-based dietary advice is composed of a 5-tier health sensitivity result (Very Low, Low, Medium, High, Very High) for Carbohydrates, Sugar, Salt, Saturated Fat, Fat, Obesity Risk, Calories and Caffeine Metabolism. These results are normally provided within the DnaNudge app, but were converted into a hard-copy document for participants in the Intervention arm of the ASPIRE-DNA study. An example of the DNA-based dietary guidelines is provided in Figure S1.

*Figure S1: Example genetic report that provides the DNA-based guidelines to participants.*

## DNA-based Dietary Guidelines: Process

Below is the process that was followed throughout the trial to determine which ‘highest risk’ genetic results to select which then formed the participant’s three goals throughout the trial.

Using the participant’s genetic results the three highest risk results were selected.

Note: Only six of the eight Genetic Report results (please see Figure S1) were considered when assessing the three highest risk results. Obesity risk and Caffeine Sensitivity were excluded from this process as; (i) the Obesity risk had already been taken into account and mapped to calories and other relevant macronutrients, and (ii) no actionable dietary intake recommendations could be made based on the Caffeine results.

**Example 1:**

In the below example, in no particular order, the three highest genetic risk results are Salt sensitivity, Carbohydrate sensitivity, and Calorie sensitivity. These formed the basis of the participant’s intervention.

See Example genetic sensitivity results below:

| Obesity Risk | Calorie Sensitivity | Fat Sensitivity | Saturated Fat Sensitivity | Carbohydrate Sensitivity | Sugar Sensitivity | Salt Sensitivity | Caffeine Metabolism |
| --- | --- | --- | --- | --- | --- | --- | --- |
| 5 | 2 | 4 | 3 | 2 | 5 | 1 | 1 |

Key for genetic results:

| **5** | very low/fast |
| --- | --- |
| **4** | low |
| **3** | medium; |
| **2** | high |
| **1** | very high/slow |

**Example 2:**

If three highest risk genetic results could not be determined i.e. there were more than three genetic results with the highest risk, the FFQ from the participant’s baseline clinical visit (visit 3) was analysed (via FETA software) and the highest intake of nutrients was selected. FETA is an open-source tool that processes dietary data from FFQs to generate a spreadsheet containing energy, nutrient, and food group intakes.

See Example dietary results below:


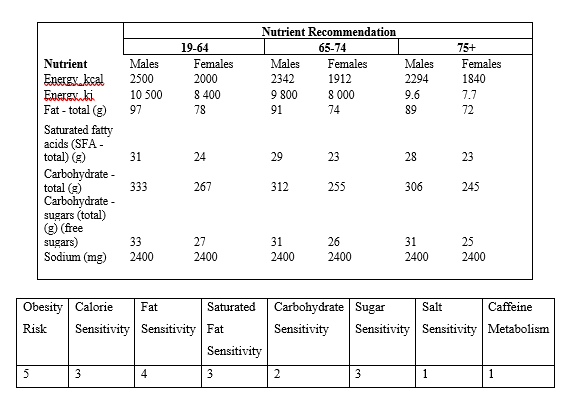


*Table S1: Description of nutrient recommendations*

From the example above, in no particular order, the first two highest risk genetic results were Salt sensitivity and Carbohydrate sensitivity. However, Sugar, Saturated fat, and Calorie sensitivity all had the same result, and only one of them needs to be selected to form the three goals. Therefore, based on baseline FFQ analysis the highest nutrient value was selected to form the third goal. For example, if calorie intake was the highest value, then the highest risk genetic results that would form the participant’s intervention would be, in no order, Salt sensitivity, Carbohydrate sensitivity and Calorie sensitivity. The determination of the “highest nutrient intake” was calculated by determining the percentage above the recommended nutrient intake based on age and sex.

Note: The Obesity Risk and Caffeine Metabolism genetic results were not applicable for the FFQ analysis.

**Reference**

Public Health England (2016). *Government Dietary Recommendations: Government recommendations for energy and nutrients for males and females aged 1-18 years and 19+ years.* Accessed at <https://assets.publishing.service.gov.uk/government/uploads/system/uploads/attachment_data/file/618167/government_dietary_recommendations.pdf>

# SNP Development Background and Information

| *Table S2: Clinical trial SNPs with location and associated trait* | | | |
| --- | --- | --- | --- |
| **SNP** | **Gene** | **Genomic location (CHR:BP, GRCh38)** | **Associated trait ^(References)^** |
| rs10811661 | *-* | 9:22134095 | T2DM  ^1–4^ |
| rs1367117 | *APOB* | 2:21041028 | Cholesterol ^5–9^ |
| rs1378942 | *CSK* | 15:74785026 | Hypertension ^10–16^ |
| rs1558902 | *FTO* | 16:53769662 | Obesity, T2DM ^5,17–28^ |
| rs2479409 | *PCSK9* | 1:55038977 | Cholesterol ^6–9,29^ |
| rs4420638 | *APOC1* | 19:44919689 | Cholesterol ^6–9,30–36^ |
| rs6065906 | *-* | 20:45925376 | Cholesterol ^6–9,29,37,38^ |
| rs6567160 | *-* | 18:60161902 | Obesity ^5,17–22,39–47^ |
| rs699 | *AGT* | 1:230710048 | Hypertension ^13,48,49^ |
| rs762551 | *CYP1A2* | 15:74749576 | Caffeine ^50,51^ |
| rs7903146 | *TCF7L2* | 10:112998590 | Obesity, T2DM ^2,17,18,20–22,30,39–41,52–76^ |
| rs8050136 | *FTO* | 16:53782363 | Obesity, T2DM ^5,52–55,77–80^ |

SNPs associated with genetic propensity for the development of obesity, T2DM, hypertension and cholesterol were selected via a rigorous process that assessed published studies, including Genome Wide Association Studies (GWAS). Additional criteria for the selection of SNPs included number of associated publications, and, for each publication, statistical significance of the variant under the canonic genome-wide significance p-value threshold of 5×10^-8^, effect of the variant such as betas or O.R., study sample size, ethnic origin of subjects (added significance given to SNPs that were studied on more than one ethnic group and were also shown to have an effect on other than European populations), sexes included in the study and effects shown on both males and females, effects shown on adults, relevant effect allele frequency, and in case of multiple variants on the same location, ensuring that the other variants were considered very rare and/or had no effect. Linkage disequilibrium was also taken into account. Based on the above, the selection of SNPs was identified; it should be noted that some of these SNPs were shown to be associated with genetic risk for more than one chronic condition. This was reflected in the assessment of the compound genetic risk for each individual condition, via the assignment of “weight factors” based on the strength of the SNP association to each condition (e.g. if the SNP affected two conditions it would be given a higher weighting factor for the condition it has a stronger association with, and a lower weighting factor for the condition it is not as strongly associated).

**References**

1. Cook, J. P. & Morris, A. P. Multi-ethnic genome-wide association study identifies novel locus for type 2 diabetes susceptibility. *Eur J Hum Genet* **24**, 1175–1180 (2016).

2. Manning, A. K. *et al.* A genome-wide approach accounting for body mass index identifies genetic variants influencing fasting glycemic traits and insulin resistance. *Nat Genet* **44**, 659–669 (2012).

3. Hwang, J. Y. *et al.* Genome-wide association meta-analysis identifies novel variants associated with fasting plasma glucose in East Asians. *Diabetes* **64**, 291–298 (2015).

4. Xue, A. *et al.* Genome-wide association analyses identify 143 risk variants and putative regulatory mechanisms for type 2 diabetes. *Nat Commun* **9**, (2018).

5. Wojcik, G. L. *et al.* Genetic analyses of diverse populations improves discovery for complex traits. *Nature* **570**, 514–518 (2019).

6. Bentley, A. R. *et al.* Multi-ancestry genome-wide gene-smoking interaction study of 387,272 individuals identifies new loci associated with serum lipids. *Nat Genet* **51**, 636–648 (2019).

7. Spracklen, C. N. *et al.* Association analyses of East Asian individuals and trans-ancestry analyses with European individuals reveal new loci associated with cholesterol and triglyceride levels. *Hum Mol Genet* **26**, 1770–1784 (2017).

8. Teslovich, T. M. *et al.* Biological, clinical and population relevance of 95 loci for blood lipids. *Nature* **466**, 707–713 (2010).

9. Willer, C. J. *et al.* Discovery and refinement of loci associated with lipid levels. *Nat Genet* **45**, 1274–1285 (2013).

10. Surendran, P. *et al.* Trans-ancestry meta-analyses identify rare and common variants associated with blood pressure and hypertension. *Nat Genet* **48**, 1151–1161 (2016).

11. Wain, L. V. *et al.* Genome-wide association study identifies six new loci influencing pulse pressure and mean arterial pressure. *Nat Genet* **43**, 1005–1012 (2011).

12. Wain, L. V. *et al.* Novel Blood Pressure Locus and Gene Discovery Using Genome-Wide Association Study and Expression Data Sets From Blood and the Kidney. *Hypertension* **70**, e4–e19 (2017).

13. Giri, A. *et al.* Trans-ethnic association study of blood pressure determinants in over 750,000 individuals. *Nat Genet* **51**, 51–62 (2019).

14. Newton-Cheh, C. *et al.* Genome-wide association study identifies eight loci associated with blood pressure. *Nat Genet* **41**, 666–676 (2009).

15. Ehret, G. B. *et al.* Genetic Variants in Novel Pathways Influence Blood Pressure and Cardiovascular Disease Risk. *Nature* **478**, 103 (2011).

16. Sung, Y. J. *et al.* A Large-Scale Multi-ancestry Genome-wide Study Accounting for Smoking Behavior Identifies Multiple Significant Loci for Blood Pressure. *Am J Hum Genet* **102**, 375–400 (2018).

17. Locke, A. E. *et al.* Genetic studies of body mass index yield new insights for obesity biology. *Nature* **518**, 197 (2015).

18. Winkler, T. W. *et al.* The Influence of Age and Sex on Genetic Associations with Adult Body Size and Shape: A Large-Scale Genome-Wide Interaction Study. *PLoS Genet* **11**, e1005378 (2015).

19. Felix, J. F. *et al.* Genome-wide association analysis identifies three new susceptibility loci for childhood body mass index. *Hum Mol Genet* **25**, 389–403 (2016).

20. Akiyama, M. *et al.* Genome-wide association study identifies 112 new loci for body mass index in the Japanese population. *Nat Genet* **49**, 1458–1467 (2017).

21. Justice, A. E. *et al.* Genome-wide meta-analysis of 241,258 adults accounting for smoking behaviour identifies novel loci for obesity traits. *Nature Communications 2017 8:1* **8**, 1–19 (2017).

22. Hoffmann, T. J. *et al.* A Large Multiethnic Genome-Wide Association Study of Adult Body Mass Index Identifies Novel Loci. *Genetics* **210**, 499–515 (2018).

23. Speliotes, E. K. *et al.* Association analyses of 249,796 individuals reveal 18 new loci associated with body mass index. *Nat Genet* **42**, 937–948 (2010).

24. Tachmazidou, I. *et al.* Whole-Genome Sequencing Coupled to Imputation Discovers Genetic Signals for Anthropometric Traits. *Am J Hum Genet* **100**, 865–884 (2017).

25. Wen, W. *et al.* Meta-analysis of genome-wide association studies in East Asian-ancestry populations identifies four new loci for body mass index. *Hum Mol Genet* **23**, 5492 (2014).

26. Warrington, N. M. *et al.* A genome-wide association study of body mass index across early life and childhood. *Int J Epidemiol* **44**, 700–712 (2015).

27. Stergiakouli, E. *et al.* Genome-wide association study of height-adjusted BMI in childhood identifies functional variant in ADCY3. *Obesity (Silver Spring)* **22**, 2252–2259 (2014).

28. Kamura, Y. *et al.* FTO Gene Polymorphism Is Associated with Type 2 Diabetes through Its Effect on Increasing the Maximum BMI in Japanese Men. *PLoS One* **11**, 165523 (2016).

29. Hoffmann, T. J. *et al.* A large electronic-health-record-based genome-wide study of serum lipids. *Nat Genet* **50**, 401–413 (2018).

30. R, S. *et al.* Genome-wide association analysis identifies loci for type 2 diabetes and triglyceride levels. *Science* **316**, 1331–1336 (2007).

31. Kim, T., Park, A. Y., Baek, Y. & Cha, S. Genome-Wide Association Study Reveals Four Loci for Lipid Ratios in the Korean Population and the Constitutional Subgroup. *PLoS One* **12**, e0168137 (2017).

32. Sandhu, M. S. *et al.* LDL-cholesterol concentrations: a genome-wide association study. *Lancet* **371**, 483–491 (2008).

33. Kathiresan, S. *et al.* Common variants at 30 loci contribute to polygenic dyslipidemia. *Nat Genet* **41**, 56–65 (2009).

34. Waterworth, D. M. *et al.* Genetic variants influencing circulating lipid levels and risk of coronary artery disease. *Arterioscler Thromb Vasc Biol* **30**, 2264–2276 (2010).

35. Willer, C. J. *et al.* Newly identified loci that influence lipid concentrations and risk of coronary artery disease. *Nat Genet* **40**, 161–169 (2008).

36. Kathiresan, S. *et al.* Six new loci associated with blood low-density lipoprotein cholesterol, high-density lipoprotein cholesterol or triglycerides in humans. *Nat Genet* **40**, 189–197 (2008).

37. Ligthart, S. *et al.* Bivariate genome-wide association study identifies novel pleiotropic loci for lipids and inflammation. *BMC Genomics* **17**, (2016).

38. Chasman, D. I. *et al.* Forty-Three Loci Associated with Plasma Lipoprotein Size, Concentration, and Cholesterol Content in Genome-Wide Analysis. *PLoS Genet* **5**, e1000730 (2009).

39. Pulit, S. L. *et al.* Meta-analysis of genome-wide association studies for body fat distribution in 694 649 individuals of European ancestry. *Hum Mol Genet* **28**, 166–174 (2019).

40. Kichaev, G. *et al.* Leveraging Polygenic Functional Enrichment to Improve GWAS Power. *Am J Hum Genet* **104**, 65–75 (2019).

41. The GIANTconsortium *et al.* Variants in the FTO and CDKAL1 loci have recessive effects on risk of obesity and type 2 diabetes, respectively. *Diabetologia* **59**, 1214–1221 (2016).

42. Pei, Y. F. *et al.* Meta-analysis of genome-wide association data identifies novel susceptibility loci for obesity. *Hum Mol Genet* **23**, 820 (2014).

43. Shungin, D. *et al.* New genetic loci link adipose and insulin biology to body fat distribution. *Nature* **518**, 187 (2015).

44. Lu, Y. *et al.* New loci for body fat percentage reveal link between adiposity and cardiometabolic disease risk. *Nat Commun* **7**, (2016).

45. Ng, M. C. Y. *et al.* Discovery and fine-mapping of adiposity loci using high density imputation of genome-wide association studies in individuals of African ancestry: African Ancestry Anthropometry Genetics Consortium. *PLoS Genet* **13**, e1006719 (2017).

46. Bradfield, J. P. *et al.* A trans-ancestral meta-analysis of genome-wide association studies reveals loci associated with childhood obesity. *Hum Mol Genet* **28**, 3327–3338 (2019).

47. Hübel, C. *et al.* Genomics of body fat percentage may contribute to sex bias in anorexia nervosa. *Am J Med Genet B Neuropsychiatr Genet* **180**, 428–438 (2019).

48. Van Der Harst, P. & Verweij, N. Identification of 64 Novel Genetic Loci Provides an Expanded View on the Genetic Architecture of Coronary Artery Disease. *Circ Res* **122**, 433–443 (2018).

49. Liu, C. *et al.* Meta-analysis identifies common and rare variants influencing blood pressure and overlapping with metabolic trait loci. *Nat Genet* **48**, 1162–1170 (2016).

50. Denden, S., Bouden, B., Haj Khelil, A., Ben Chibani, J. & Hamdaoui, M. H. Gender and ethnicity modify the association between the CYP1A2 rs762551 polymorphism and habitual coffee intake: evidence from a meta-analysis. *Genet Mol Res* **15**, (2016).

51. Pataky, M. W. *et al.* Caffeine and 3-km cycling performance: Effects of mouth rinsing, genotype, and time of day. *Scand J Med Sci Sports* **26**, 613–619 (2016).

52. Graff, M. *et al.* Genome-wide physical activity interactions in adiposity - A meta-analysis of 200,452 adults. *PLoS Genet* **13**, (2017).

53. Zhao, W. *et al.* Identification of new susceptibility loci for type 2 diabetes and shared etiological pathways with coronary heart disease. *Nat Genet* **49**, 1450–1457 (2017).

54. Timpson, N. J. *et al.* Adiposity-Related Heterogeneity in Patterns of Type 2 Diabetes Susceptibility Observed in Genome-Wide Association Data. *Diabetes* **58**, 505 (2009).

55. Scott, L. J. *et al.* A genome-wide association study of type 2 diabetes in Finns detects multiple susceptibility variants. *Science* **316**, 1341–1345 (2007).

56. Keaton, J. M. *et al.* Genome-wide interaction with the insulin secretion locus MTNR1B reveals CMIP as a novel type 2 diabetes susceptibility gene in African Americans. *Genet Epidemiol* **42**, 559 (2018).

57. Steinthorsdottir, V. *et al.* A variant in CDKAL1 influences insulin response and risk of type 2 diabetes. *Nat Genet* **39**, 770–775 (2007).

58. Hackinger, S. *et al.* Evidence for genetic contribution to the increased risk of type 2 diabetes in schizophrenia. *Transl Psychiatry* **8**, (2018).

59. Qi, Q. *et al.* Genetics of type 2 diabetes in U.S. Hispanic/Latino individuals: Results from the Hispanic Community Health Study/Study of Latinos (HCHS/SOL). *Diabetes* **66**, 1419–1425 (2017).

60. Takeuchi, F. *et al.* Confirmation of Multiple Risk Loci and Genetic Impacts by a Genome-Wide Association Study of Type 2 Diabetes in the Japanese Population. *Diabetes* **58**, 1690 (2009).

61. Chen, J. *et al.* Genome-wide association study of type 2 diabetes in Africa. *Diabetologia* **62**, 1204–1211 (2019).

62. Williams Amy, A. L. *et al.* Sequence variants in SLC16A11 are a common risk factor for type 2 diabetes in Mexico. *Nature* **506**, 97–101 (2014).

63. Hara, K. *et al.* Genome-wide association study identifies three novel loci for type 2 diabetes. *Hum Mol Genet* **23**, 239–246 (2014).

64. Kho, A. N. *et al.* Use of diverse electronic medical record systems to identify genetic risk for type 2 diabetes within a genome-wide association study. *J Am Med Inform Assoc* **19**, 212–218 (2012).

65. Zeggini, E. *et al.* Meta-analysis of genome-wide association data and large-scale replication identifies additional susceptibility loci for type 2 diabetes. *Nat Genet* **40**, 638–645 (2008).

66. Rung, J. *et al.* Genetic variant near IRS1 is associated with type 2 diabetes, insulin resistance and hyperinsulinemia. *Nat Genet* **41**, 1110–1115 (2009).

67. Sladek, R. *et al.* A genome-wide association study identifies novel risk loci for type 2 diabetes. *Nature* **445**, 881–885 (2007).

68. Tabassum, R. *et al.* Genome-wide association study for type 2 diabetes in Indians identifies a new susceptibility locus at 2q21. *Diabetes* **62**, 977–986 (2013).

69. Perry, J. R. B. *et al.* Stratifying Type 2 Diabetes Cases by BMI Identifies Genetic Risk Variants in LAMA1 and Enrichment for Risk Variants in Lean Compared to Obese Cases. *PLoS Genet* **8**, e1002741 (2012).

70. Flannick, J. *et al.* Exome sequencing of 20,791 cases of type 2 diabetes and 24,440 controls. *Nature 2019 570:7759* **570**, 71–76 (2019).

71. Voight, B. F. *et al.* Twelve type 2 diabetes susceptibility loci identified through large-scale association analysis. *Nat Genet* **42**, 579–589 (2010).

72. Saxena, R. *et al.* Genome-wide association study identifies a novel locus contributing to type 2 diabetes susceptibility in Sikhs of Punjabi origin from India. *Diabetes* **62**, 1746–1755 (2013).

73. Mahajan, A. *et al.* Genome-wide trans-ancestry meta-analysis provides insight into the genetic architecture of type 2 diabetes susceptibility. *Nat Genet* **46**, 234–244 (2014).

74. Ng, M. C. Y. *et al.* Meta-Analysis of Genome-Wide Association Studies in African Americans Provides Insights into the Genetic Architecture of Type 2 Diabetes. *PLoS Genet* **10**, e1004517 (2014).

75. Morris, A. P. *et al.* Large-scale association analysis provides insights into the genetic architecture and pathophysiology of type 2 diabetes. *Nat Genet* **44**, 981–990 (2012).

76. Strawbridge, R. J. *et al.* Genome-wide association identifies nine common variants associated with fasting proinsulin levels and provides new insights into the pathophysiology of type 2 diabetes. *Diabetes* **60**, 2624–2634 (2011).

77. Thorleifsson, G. *et al.* Genome-wide association yields new sequence variants at seven loci that associate with measures of obesity. *Nat Genet* **41**, 18–24 (2009).

78. Zeggini, E. *et al.* Replication of genome-wide association signals in UK samples reveals risk loci for type 2 diabetes. *Science* **316**, 1336–1341 (2007).

79. Chiang, K. M. *et al.* Genome-wide association study of morbid obesity in Han Chinese. *BMC Genet* **20**, (2019).

80. Wan, E. S. *et al.* Genome-wide association analysis of body mass in chronic obstructive pulmonary disease. *Am J Respir Cell Mol Biol* **45**, 304–310 (2011).
